# Temozolomide‐Promoted MGMT Transcription Contributes to Chemoresistance by Activating the ERK Signalling Pathway in Malignant Melanoma

**DOI:** 10.1111/jcmm.70380

**Published:** 2025-01-28

**Authors:** Meiyi Deng, Bingjie Ren, Jing Zhao, Xia Guo, Yuanyuan Yang, Huiling Shi, Xuyu Bian, Mengyao Wu, Caihua Xu, Min Tao, Rongrui Liang, Qiang Li

**Affiliations:** ^1^ Department of Oncology The Fourth Affiliated Hospital of Soochow University Suzhou Jiangsu China; ^2^ Division of Clinical Oncology Medical Center of Soochow University Suzhou Jiangsu China; ^3^ Suzhou Sano Precision Medicine Ltd Suzhou Jiangsu China; ^4^ Department of Oncology The First Affiliated Hospital of Soochow University Suzhou Jiangsu China; ^5^ Department of Oncology Nanyang Second General Hospital Nanyang Henan China; ^6^ Department of Radiation Oncology The First Affiliated Hospital of Soochow University Suzhou Jiangsu China; ^7^ Department of Pathology The First Affiliated Hospital of Soochow University Suzhou Jiangsu China; ^8^ Department of Chemotherapy Jiangxi Cancer Hospital Nanchang Jiangxi China

**Keywords:** chemoresistance, MAPK/ERK pathway, melanoma, MGMT, temozolomide

## Abstract

Tumour cells possess a multitude of chemoresistance mechanisms, which could plausibly contribute to the ineffectiveness of chemotherapy. O^6^‐methylguanine‐DNA methyltransferase (MGMT) is an important effector protein associated with Temozolomide (TMZ) resistance in various tumours. To some extent, the expression level of MGMT determines the sensitivity of cells to TMZ, but the mechanism of its expression regulation has not been fully elucidated. Cultured malignant melanoma cell lines A375 and Sk‐MEL28 were employed. A luciferase assay was used to detect the transcriptional activity of the MGMT promoter. Western blotting was used to compare the expression levels of phosphorylated ERK1/2 (P‐ERK1/2) after TMZ treatment. Immunofluorescent staining was used to detect TMZ‐induced DNA damage protein levels. The sensitivity of melanoma cells to TMZ was detected by MTT assay and animal experiments. The expression of MGMT mRNA was tested by Quantitative real‐time PCR (RT‐qPCR). Flow cytometry was used to measure the apoptosis of TMZ‐treated cells. TMZ enhanced the transcription of MGMT through activating the ERK pathway. ERK inhibitors U0126 and vemurafenib (vMF) inhibited the TMZ induced transcription of MGMT. The expression of MGMT and p‐ERK1/2 was closely related in human MM tissues. vMF increased the sensitivity of melanoma (MM) to TMZ in vitro and in vivo through downregulating MGMT and promoting the TMZ induced DNA damage in MM. TMZ‐promoted MGMT transcription contributed to instinctive chemoresistance by activating the ERK signalling pathway in malignant melanoma. Our study indicates that the use of the ERK inhibitor in combination with TMZ could potentially enhance the effectiveness of clinical treatment for malignant melanoma.

## 1 Introduction

Malignant melanoma results from the malignant transformation of melanocytes, which are of neural crest origin [1, 2, 3]. Melanoma mostly occurs in the skin, but can also be seen in mucous membranes and internal organs. Melanoma at early stages can be cured by the surgical resection of the primary lesion. Advanced melanomas undergo systemic therapy, which includes chemotherapy, targeted therapy and immunotherapy. Among them, TMZ is a commonly used chemotherapy drug for the treatment of advanced melanoma. Clinical studies have shown that TMZ is more effective than dacarbazine (DTIC) in advanced melanoma [4]. Recently, the US Food and Drug Administration approved TIL therapy for the treatment of patients with unresectable or metastatic melanoma. Adoptive cell therapies are currently being tested in combination with other therapies, personalised melanoma vaccines, modified immune cells or drugs that target immune checkpoints. Among them, the use of oncolytic virus [219220] or mRNA‐based melanoma vaccines in melanoma treatment deserves special attention [5, 6, 7, 8]. In addition, vitamin D supplementation has been reported to improve the objective response rate and prolong progression‐free time in melanoma patients receiving anti–PD‐1 therapy [9], and vitamin D supplementation may reduce melanoma cases [10]. There was a recent review that outlined how vitamin D signalling, defined by selective activation of specific nuclear receptors and specific vitamin D hydroxyl derivatives, can provide benefits for new or existing treatments [11], which opens a window of opportunity to augment existing treatments with simple nutritional product supplementation.

Although there has been a lot of drug research on melanin therapy this year, the utility of these strategies has been limited to some extent due to adverse effects, financial costs, and inherent or acquired tumour resistance mechanisms that lead to the recurrence and death of patients, and the prognosis of malignant melanoma remains poor, with only about 30% of malignant melanoma cells showing a transient clinical response to chemotherapy. Therefore, it is particularly important to unravel the complex mechanistic basis of chemoresistance and develop effective treatments. Tumour cells possess a multitude of chemoresistance mechanisms, which could plausibly contribute to the ineffectiveness of chemotherapy, Ultimately leading to the failure of anti‐cancer treatment [12, 13]. Malignant melanoma (MM) is a highly aggressive cancer with a poor prognosis and a high incidence of chemoresistance. YAP1 controls the N‐cadherin–mediated tumour–stroma interaction in melanoma progression. The primary mechanism underlying chemoresistance in MM is through the action of the DNA repair protein O^6^‐methylguanine DNA methyltransferase (MGMT). MGMT effectively eliminates the alkylation damage at the O6 position of guanine and repairs cytotoxic damage induced by alkylating agents, such as cyclophosphamide (CTX), dacarbazine (DTIC) and temozolomide (TMZ), leading to drug resistance [14, 15]. TMZ is a classical chemo‐drug widely administered in MM treatment. It was reported that TMZ could penetrate the blood–brain barrier; therefore, it is also used for the treatment of brain metastases of melanoma [16]. Several mechanisms of TMZ resistance associated with MGMT have been reported in the relevant literature. Besides, abnormal levels of mismatch repair (MMR) proteins were also reported in metastatic melanoma [17], downregulation or impaired MMR might be considered a cause of acquired resistance to TMZ and other methylating drugs (together with MGMT). Moreover, cytokines are also involved in the resistance of melanoma cells to TMZ. It was reported that the tumour suppressor cytokine IL‐24 is downregulated during melanoma progression, and supplementing IL‐24 has been shown to reverse melanoma resistance to TMZ by downregulating MGMT [18]. In addition, a study revealed that receptor interacting protein 2 (RIP2) was upregulated in TMZ‐resistant glioma cells in correlation with drug resistance, and the RIP2/NF‐κB/MGMT signalling pathway plays a vital role in the regulation of TMZ resistance [19]. Another study showed that TGF‐β1 decreased miR‐198 levels and increased MGMT accumulation to confer TMZ resistance [20]. Nonetheless, glioblastoma (GBM) cells with low levels of MGMT still show resistance to TMZ, suggesting that MGMT‐independent mechanisms are also involved in the initial or acquired resistance to TMZ [21]. Taken together, most studies have proven that the main resistance of tumour cells to TMZ is the expression level of MGMT. Hence, MGMT expression might be regulated by multiple molecular mechanisms. However, a mechanism of MGMT expression regulation still needs to be elucidated. Compared to other types of cancer, melanoma expresses quite low levels of MGMT, similar to malignant glioma [22], which might explain why melanomas respond to the methylating drugs DTIC and TMZ. Therefore, we believe that inhibiting the expression level of MGMT protein can reduce chemoresistance.

In the past, the systemic clinical application of MGMT inhibitors has been restricted mainly because of an increase in the haematological toxicity of DNA alkylators and failure to restore TMZ sensitivity in TMZ‐resistant malignancies. Even so, the selective inhibitors of the BRAF oncogene, alone or in combination with MEK inhibitors, have achieved a response rate of approximately 50%–70%, resulting in improved progression‐free survival (PFS) and overall survival (OS), as shown in phase III studies of patients harbouring BRAF mutations [23, 24]. Relevant studies indicate that the MAPK signalling pathway may be associated with the overexpression of MGMT, which may be a potential way to obtain TMZ resistance [25, 26]. But the exact mechanism has not been elaborated. Hence, in this study, we investigated the mechanism that TMZ promotes transcription of the MGMT promoter by activating the ERK signalling pathway. We believe that this may be related to acquired drug resistance, and some cells that were originally sensitive to TMZ may develop chemotherapy resistance by promoting MGMT transcription. Furthermore, we also identified the effect of the ERK inhibitors on sensitising MM cells to TMZ, which may have guiding significance for the treatment of clinical TMZ.

## 2 Materials and Methods

### 2.1 Cell Lines and Culture

A375 and Sk‐MEL28 are BRAF V600E mutant human malignant melanoma cells obtained from the Guangzhou National Center Cell Bank (NECB). The cells were cultured in DMEM (Thermo Scientific, Waltham, MA, USA) containing 10% FBS (Thermo Scientific). Cells were incubated at 37°C with 5% CO_2_.

### 2.2 Animals

Male BALB/c nude mice (4 weeks old) were obtained from the Nanjing Experimental Animal Center and maintained in the laboratory animal centre of Soochow University following the guidelines of the Soochow University Institutional Animal Care and Use Committee. The animal experimental research protocol was approved by the Medical Ethics Committee (No. 2023 LS(Application) Approval No. 23003). A total of 1 × 10^6^ A375 cells resuspended in 200 μL of PBS were subcutaneously injected under the armpits of male nude mice (5 weeks old). Tumours were visible in all mice. The mice were randomly separated into three groups (*n* = 6): group I, treatment with DMSO; group II, treatment with 20 mg/kg·d TMZ; and group III, treatment with 20 mg/kg·d TMZ and 20 mg/kg·d vemurafenib. The treatments were performed 10 days after the injection of A375 cells. Tumours were measured with Vernier callipers every 2 days, and tumour volumes were calculated using the formula volume = (length × width^2^)/2 and reported as the mean size ± standard error.

### 2.3 MGMT‐Overexpressing Plasmids and Transfection

The pcDNA3.1‐MGMT OE vector was produced by Sangon Biotech (Shanghai) Co. Ltd. The plasmid sequence was verified by DNA sequencing supplemental data. Cells (1 × 10^5^) were seeded in each well of six‐well plates 24 h before transfection. Lipofectamine 3000 (Thermo Fisher Scientific Inc.) was utilised to perform transfection with 5.0 μg of the pcDNA3.1(+)‐MGMT vector or 5.0 μg of the pcDNA3.1(+) empty vector (as a negative control) according to the manufacturer's instructions.

### 2.4 Luciferase Reporter Plasmid and Luciferase Assay

The MGMT promoter fragment (1.0 kb) was ligated into the equivalent site of the pGL4.84‐basic vector (Promage, Beijing, China) to form the MGMT promoter‐luciferase reporter construct, designated as MGMT‐Luc. Cells were seeded in six‐well plates and grown to 70%–80% confluence; then, each well contained 5 × 10^5^ cells and 5.0 μg MGMT‐Luc, according to the manufacturer's instructions for Lipofectamine 3000 (Thermo Fisher Scientific Inc.). All the cells underwent the luciferase reporter assay 36 h after the completion of the transfection procedure. The activities of Renilla luciferase in MGMT‐Luc were determined following the Renilla Luciferase Reporter Gene Assay Kit protocol (Beyotime, RG017) as previously reported [27]. After the intervention, the cells were rinsed with PBS before harvesting, and 100 μL cell lysates per well were prepared in six‐well plates. 100 μL of cell lysate was transferred into a 96‐well cell culture (black) containing 100 μL of Renilla luciferase detection working fluid. The reading was performed by a multifunction enzyme marker (Tecan Spark, Switzerland).

### 2.5 Assessment of Cell Viability and Apoptosis

Cell viability was detected using the MTT assay as previously reported [25]. After the treatment of cells with the indicated agent for 24, 48 or 72 h, the cells were rinsed two times with PBS and incubated with MTT (0.5 mg/mL; Sigma) for 4 h. The reagent was absorbed by living cells and ultimately formed an insoluble blue formazan product. The formazan product was solubilised with DMSO and quantified using a microplate reader by measuring the absorbance at 550 nm. The inhibition rate and IC_50_ were calculated utilising SPSS software (version 26.0, SPSS Inc., Chicago, IL, USA). The growth inhibition rate was calculated as follows: growth inhibition rate = (1‐A570 value of the drug‐treated group/A570 value of the control group) × 100%.

### 2.6 Detection of Cell Apoptosis

The apoptosis ratio was evaluated with the Annexin V‐FITC/PI Apoptosis Detection Kit (Signalway Antibody, College Park, MD, USA). A375 cells were seeded in six‐well plates and treated with TMZ and vMF, either alone or in combination, for 12 h. The cells were washed twice with PBS and resuspended in 400 μL of 1× binding buffer. Annexin V‐FITC A (5 μL) was added to the cell suspension, which was subsequently gently mixed and incubated for 15 min at 2°C–8°C in the dark. Then, 10 μL of PI was added, followed by mixing for 5 min at 2°C–8°C in the dark. Analysis of apoptotic cells was performed by flow cytometry (Beckman, CA, USA).

### 2.7 Combination Index (CI)

The growth inhibitory effect of different doses of individual and combination treatments was measured by assessing cell viability as described above [28]. The Chou–Talalay method for determining the effect of a drug combination is based on the median‐effect equation for determining drug interactions, where CI < 1, =1 and > 1 indicate synergism, an additive effect and antagonism, respectively. The CI value was generated using Compusyn computer software (Biosoft, Cambridge, UK) with the CI equation.

### 2.8 Western Blotting

Western blotting was performed as previously described [25]. Whole‐cell extracts were prepared from A375 cells incubated in six‐well plates. After incubation, the cells were collected, rinsed with PBS and lysed in extraction buffer (40 mmol/L Tris–HCl, pH 7.5, 150 mmol/L KCl, 1 mmol/L EDTA, 1% Triton X‐100, 100 mmol/L NaVO_3_ and 1 mmol/L PMSF) supplemented with a protease inhibitor cocktail. The obtained proteins (50 μg) were separated on SurePAGE Bis‐Tris gradient concentration (4%–20%) SDS–PAGE gels (Genscript Biotech Corporation, Nanjing, China) and transferred to PVDF membranes. Membranes were blocked with 5% nonfat milk for 2 h at room temperature and then incubated with a rabbit anti–MEK1 antibody (CST, #9122), rabbit anti–p‐MEK1 antibody (CST, #9154), mouse anti–ERK1/2 antibody (CST, #4695), rabbit anti–p‐ERK1/2 antibody (CST, #4370), rabbit anti–p‐Chk1 antibody (CST, #2348), rabbit anti–p‐Chk2 antibody (CST, #82,263), rabbit anti–p‐ATR antibody (CST, #30,632), rabbit anti–p‐ATM antibody (CST, #6966), rabbit anti–γ‐H2AX (abcam, ab2893) antibody, rabbit anti–MGMT antibody (CST, #2739) or mouse anti–β‐actin antibody (CST, #8457) for 12 h at 4°C. Horseradish peroxidase–conjugated anti–mouse IgG (CST, #7076) or horseradish peroxidase–conjugated anti–rabbit IgG (CST, #7074) was used as a secondary antibody (at room temperature for 2 h), and antigen–antibody complexes were detected utilising an enhanced chemiluminescence kit (ECL Plus, Amersham, Freiburg, Germany). Densitometry values for Western blot and antibody array experiments were estimated with ImageQuant TL software (GE Healthcare, Buckinghamshire, UK) and reported as arbitrary units (a.u.). Multiple film exposures were obtained to verify the linearity of the samples analysed.

### 2.9 Quantitative Real‐Time PCR

The mRNA expression of target genes was detected by quantitative real‐time PCR using sybr green dye. A375 cells were plated in six‐well plates and treated with the designated reagents. After incubation, the cells were collected and total RNA was isolated utilising TRIzol reagent (Invitrogen, USA). Reverse transcription was performed with the ‘5× all in one’ RT reagent (Abm, Canada). Quantitative PCR was conducted using EvaGreen Master Mixes (Abm, Canada) according to the manufacturer's instructions. The β‐actin gene was selected as an endogenous control. The primers for MGMT were as follows: forward primer, 5′‐GTT TTC CAG CAA GAG TCG TTC‐3′ and reverse primer, 5′‐GCT GCT AAT TGC TGG TAA GAA A‐3′. The primers for β‐actin were as follows: forward primer, 5′‐CCT GGC ACC CAG CAC AAT‐3′ and reverse primer, 5′‐GGG CCG GAC TCG TCA TAC‐3′.

### 2.10 Immunofluorescence Staining

Pre‐sterilised coverslips were placed in six‐well plates, and A375 cells were seeded on the coverslips. After treatment with the designated drugs, cells were fixed with 4% paraformaldehyde for 30 min. Cells were permeabilised with a 0.1% Triton X‐100 solution for 15 min and blocked with 5% BSA for 1 h. The cells were immunostained overnight using the primary γ‐H2AX antibody (CST; 1:100) at 4°C. Then, the cells were incubated with the CoraLite 594‐conjugated goat anti‐rabbit IgG secondary antibody (Proteintech; 1:400) diluted in TBS mixed with DAPI (1, 10,000) for 1 h at room temperature in the dark. Cells were visualised using a fluorescence microscope (IX5 Observer Inverted Microscope; Olympus, Tokyo, Japan).

### 2.11 Statistical Analysis

GraphPad Prism 9 was used for statistical analysis. Differences between treatment groups were analysed by one‐way ANOVA or Student's *t*‐test. A *p* value < 0.05 was considered statistically significant. Data are presented as the means ± standard errors from at least three independent experiments.

## 3 Results

### 3.1 TMZ Enhances the Transcriptional Activity of the MGMT Promoter in MM Cells

**FIGURE 1:**
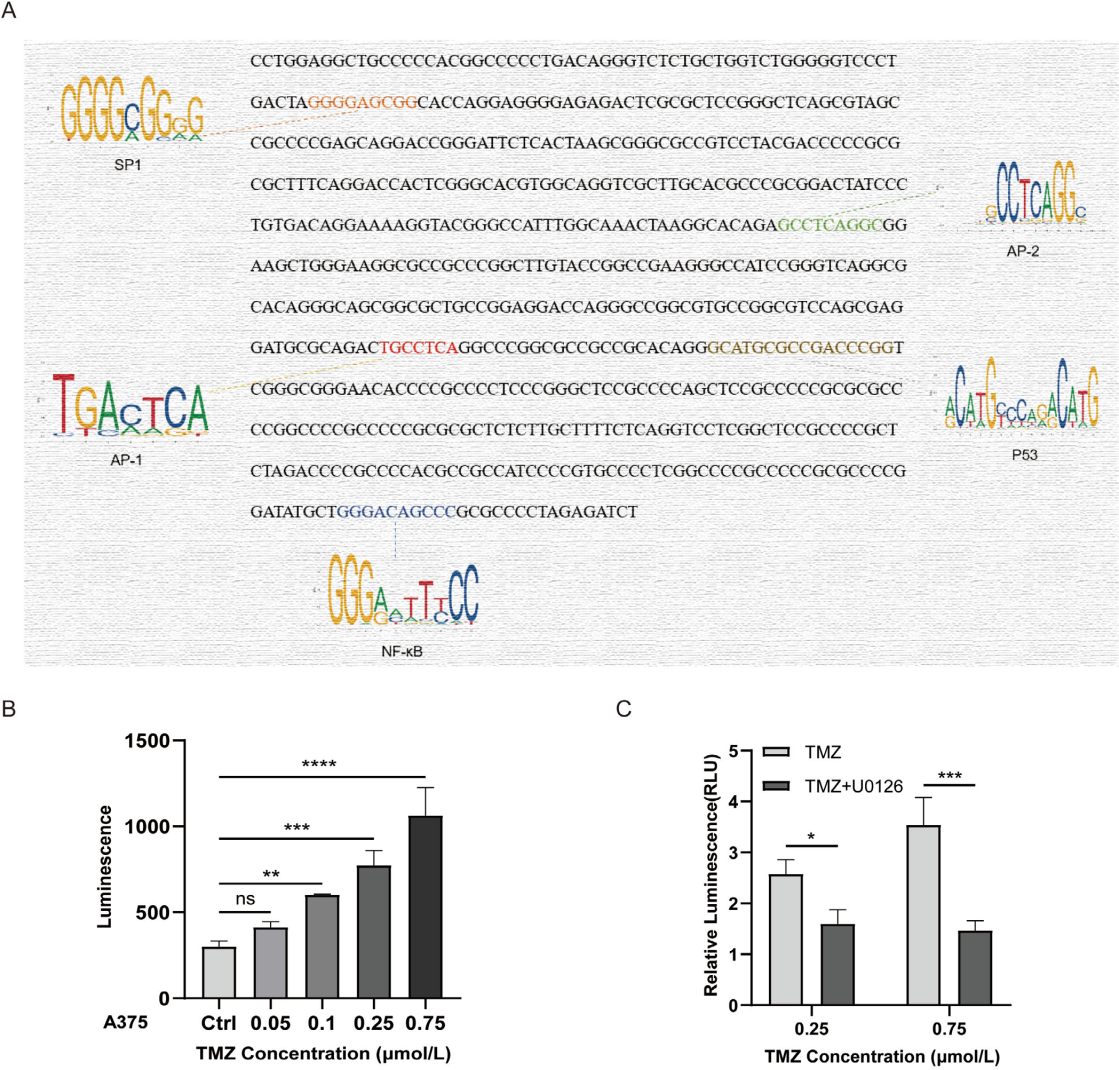
TMZ enhances the transcriptional activity of the MGMT promoter in MM cells. (A) Predicted transcription factor binding sites on the MGMT promoter (SP1, AP‐1, AP‐2, P53, NF‐κB). (B) Luciferase reporter assay to measure MGMT promoter activity. A375 cells were treated with TMZ (0.05, 0.1, 0.25 and 0.75 μM) for 12 h, and the luciferase activity was measured. Data were analysed using one‐way ANOVA (**p < 0.01, ***p < 0.001 and ****p < 0.0001 compared with the control group). (C) A375 cells were treated with 50 μM U0126 and/or TMZ (0.25, 0.75 μM) for 12 h, RLU was measured by Renilla luciferase activity. Data were analysed using one‐way ANOVA (*p < 0.05, ***p < 0.001 compared with the control group).

To identify the potential transcription factor‐binding site, we performed a sequence analysis using the JASPAR website (http://jaspar.genereg.net/). The prediction results showed that the 5′ potential MGMT promoter region contained several potential transcription factor‐binding sites, including binding sites of SP1, AP‐1, AP‐2, NF‐κB and P53 (Figure 1A). The transcriptional activity of the MGMT promoter was observed by transfection of the luciferase reporter plasmid in A375 cells with different concentrations of TMZ. After transfection of the luciferase reporter plasmid for 24 h, A375 cells were treated with 0.05, 0.1, 0.25 and 0.75 μM TMZ, and the expression of luciferase was detected. The transcriptional activity of the MGMT promoter can be reflected by luciferase expression (Figure 1B). We suggest that MGMT, as a damage repair protein, is largely depleted after TMZ action, but at the same time, TMZ stimulation also achieved a balance by promoting the transcriptional activity of the MGMT promoter to compensate for depleted MGMT. Further experiments found that the application of U0126, an inhibitor of the ERK pathway, reduced the stimulatory effect of TMZ on the transcriptional activity of the MGMT promoter (Figure 1C). Therefore, TMZ may promote MGMT transcription by activating the ERK signalling pathway through transcription factors.

### 3.2 TMZ Activates the ERK Pathway in Melanoma Cells

**FIGURE 2:**
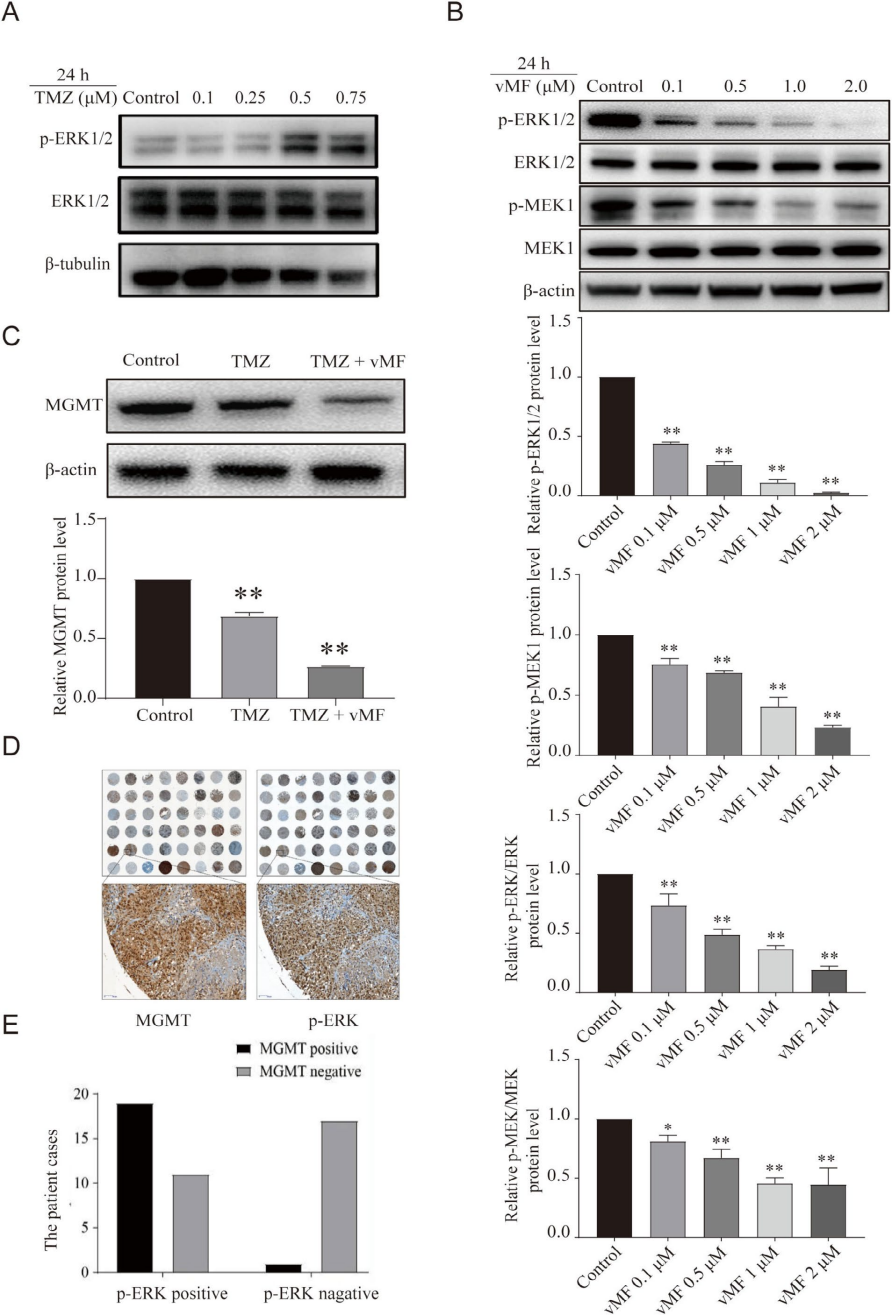
TMZ activates the ERK pathway in melanoma cells. (A) Levels of p‐ERK1/2 were detected in A375 cells treated with TMZ (0.1, 0.25, 0.5 and 0.75 μM) for 24 h using Western blot analysis. (B) Levels of p‐ERK1/2 and p‐MEK1 were detected in A375 cells treated with vMF (0.1, 0.5, 1.0 and 2.0 μM) for 24 h using Western blot analysis (*p < 0.05 and **p < 0.01 compared with the control group). (C) The levels of MGMT in MM tissues from nude mice were analysed using Western blotting (*p < 0.05 and **p < 0.01 compared with the control group). (D, E) The panels show immunohistochemical tissue staining in malignant melanoma.

After A375 cells were treated with 0.1, 0.25, 0.5 and 0.75 μM TMZ, Western blotting was used to detect the expression of p‐ERK protein in cells (Figure 2A). It was observed that the expression level of p‐ERK protein was upregulated by TMZ at higher concentrations. In contrast, vMF, an ERK inhibitor targeted at B‐RAF, inhibits the MAPK/ERK signalling pathway and reduces MGMT protein levels in cells treated with TMZ. The ERK1/2 (p‐ERK1/2) and MEK1 (p‐MEK1) proteins were detected by western blotting in A375 cells treated with vMF (0.1, 0.5, 1.0 and 2.0 μM) for 24 h (Figure 2B). Western blotting showed that the levels of p‐ERK1/2 and p‐MEK1 in the control group, 0.1 μM vMF group, 0.5 μM vMF group, 1.0 μM vMF group and 2.0 μM vMF group decreased sequentially. Therefore, we further explored the effect of TMZ combined with vMF on the expression of MGMT in vivo. Melanoma tissues were dissected from the nude mice, and total protein was extracted. The levels of MGMT were detected in the control, TMZ and TMZ + vMF groups using Western blotting (Figure 2C). The levels of MGMT decreased in the control, TMZ and TMZ + vMF groups in that order (Figure 2C).

To further determine the relationship between the ERK pathway and the MGMT protein, we executed tissue microarray experiments and found that MGMT‐positive cases were high in the p‐ERK‐positive group and low in the p‐ERK‐negative group (Figure 2D,E). We found that the levels of MGMT and p‐ERK1/2 were positively correlated in melanoma tissues. Our result further elucidates the significant relationship between the ERK signalling pathway and the transcription of MGMT. It is reasonable to believe that TMZ might promote the transcription of MGMT by activating the ERK pathway.

### 3.3 TMZ In Conjunction With vMF Downregulates MGMT Expression in Melanoma Cells

**FIGURE 3:**
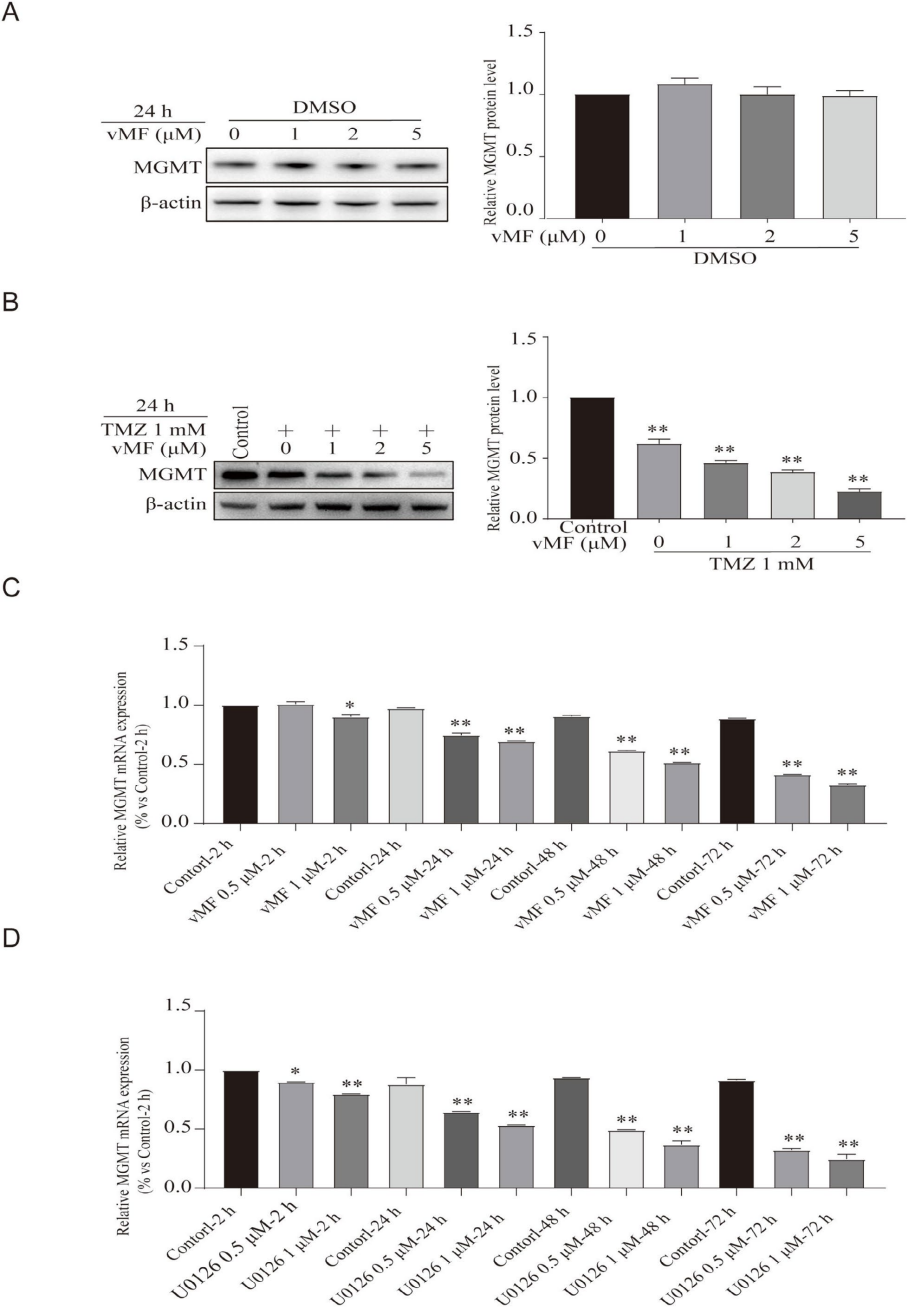
TMZ in conjunction with vMF downregulates MGMT expression in melanoma cells. (A) The expression of the MGMT protein was detected in A375 cells treated with DMSO and vMF (0, 1, 2 and 5 μM) for 24 h using Western blot analysis. (B) The expression of the MGMT protein was detected in A375 cells treated with TMZ (1 mM) and vMF (0, 1, 2 and 5 μM) for 24 h using Western blot analysis (*p < 0.05 and **p < 0.01 compared with the control group). (C) RT–qPCR analysis of MGMT mRNA expression in A375 cells treated with vMF (0.5 and 1 μM) for 2, 24, 48 and 72 h (*p < 0.05 and **p < 0.01 compared with the control‐2 h group). (D) RT–qPCR analysis of MGMT mRNA expression in A375 cells treated with U0126 (0.5 and 1 μM) for 2, 24, 48 and 72 h (*p < 0.05 and **p < 0.01 compared with the control‐2 h group).

vMF alone could not decrease the MGMT protein in melanoma cells (Figure 3A). The expression of the MGMT protein was detected in A375 cells treated with vMF (0, 1, 2 and 5 μM) and/or TMZ (1 mM) for 24 h. As shown in Figure 3B vMF reduced the level of the MGMT protein in melanoma cells exposed to TMZ in a dose‐dependent manner. This result was not consistent with the previous literature [29]. So we speculated that vMF can only decrease the level of the MGMT protein under TMZ‐induced ERK activation. We subsequently detected the expression of MGMT mRNA in A375 cells treated with vMF (0, 0.5 and 1 μM) (Figure 3C) or U0126 (0, 0.5 and 1 μM) (Figure 3D) for different times using RT‐qPCR. Based on the RT‐qPCR results, the expression of MGMT mRNA was significantly decreased with increasing vMF or U0126 concentrations. Together with the protein and mRNA data, vMF or U0126 inhibits MGMT mRNA transcription without altering the levels of the MGMT protein.

### 3.4 Overexpression of MGMT Attenuates ERK Inhibitor–Induced TMZ Sensitisation

**FIGURE 4:**
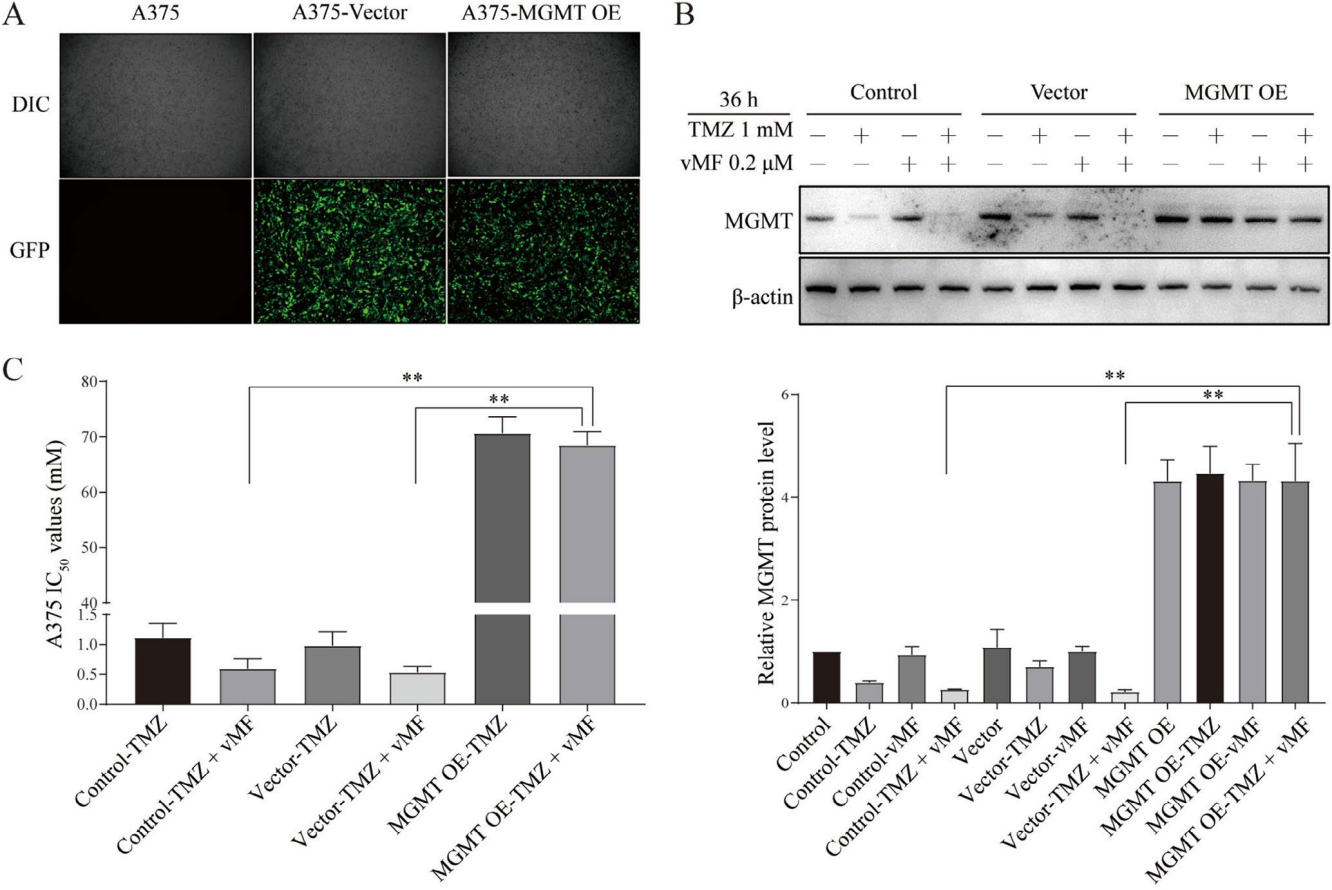
Overexpression of MGMT attenuates ERK inhibitor‐induced TMZ sensitization. (A) After transfection of the pcDNA3.1‐MGMT plasmid or the pcDNA3.1 empty vector for 24 h, the expression of the MGMT‐GFP was observed in A375 cells using fluorescence microscopy (representative fields at ×40 magnification are shown). (B) A375 cells were treated with TMZ (1 mM) and/or vMF (0.2 μM) for 36 h after transfection with the pcDNA3.1‐MGMT plasmid or the pcDNA3.1 empty vector. Western blot analysis of MGMT protein levels (*p < 0.05 and **p < 0.01 compared with the indicated treatment). (C) A375 cells were treated with TMZ and 0.04 μM vMF for 24 h after transfection with the pcDNA3.1‐MGMT plasmid or the pcDNA3.1 empty vector for 24 h. The cell inhibition rate was determined with the MTT assay. The IC50 value was calculated with SPSS software (**p < 0.01 compared with the indicated treatment).

We conducted the experiment described below to explore whether vMF increases the cytotoxicity of TMZ to MM by reducing MGMT levels. First, MGMT expression was upregulated in A375 cells by transfection with the pcDNA3.1‐MGMT plasmid (Figure 4A). Twenty‐four hours after transfection, A375 MGMT OE cells were treated with 0.2 μM vMF and/or 1 mM TMZ for 36 h, and the expression of MGMT was detected. Western blotting showed that MGMT expression in the MGMT OE‐TMZ + vMF group was upregulated than that in the control‐TMZ + vMF group and vector‐TMZ + vMF group (Figure 4B). Furthermore, the results of MTT assays showed a significant increase in IC_50_ value in the MGMT OE group compared with that of the control and vector groups (Figure 4C). Our results revealed that MGMT overexpression attenuates vMF‐induced TMZ sensitisation.

### 3.5 ERK Inhibitor Increases the Sensitivity of MM Cells to TMZ In Vitro and In Vivo

**FIGURE 5:**
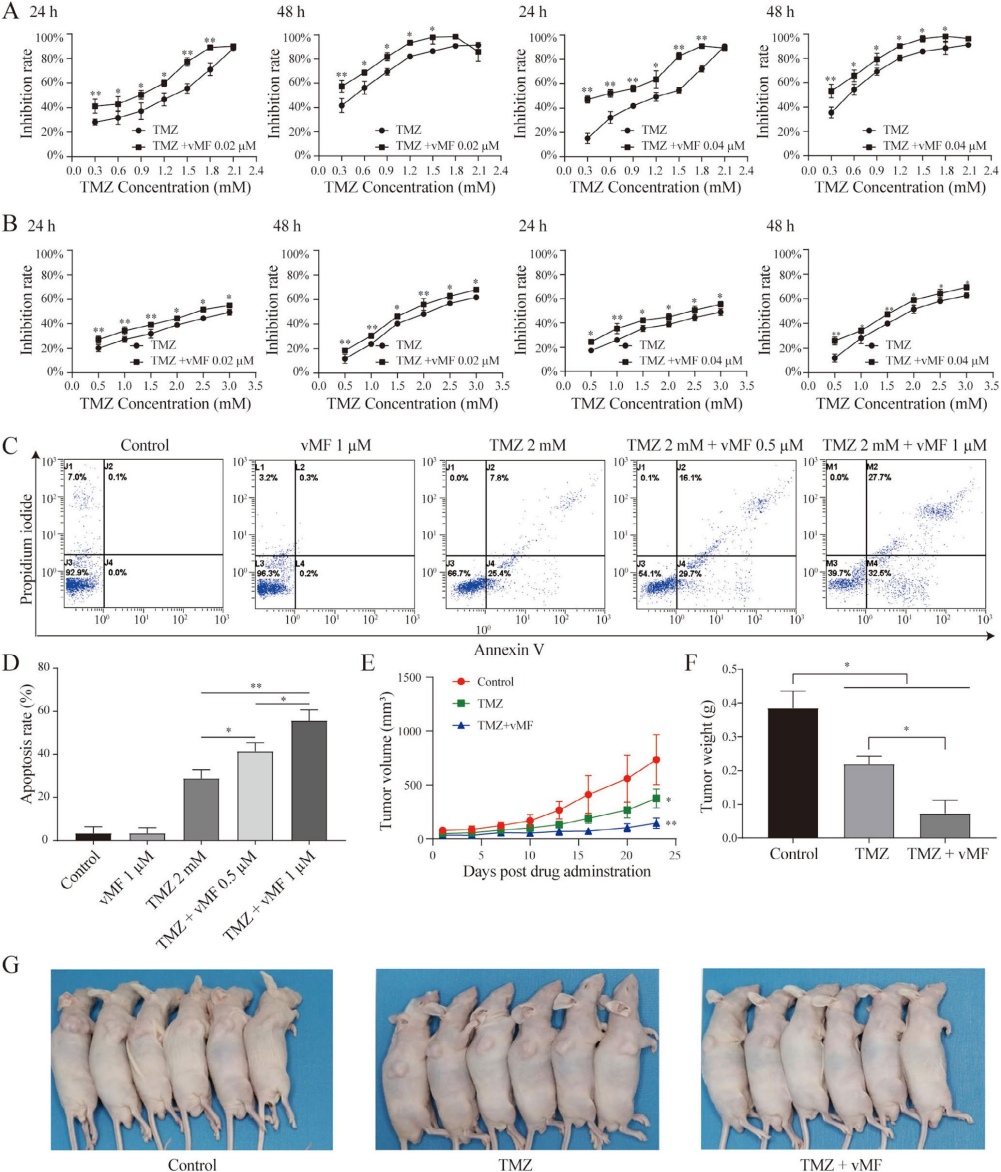
ERK inhibitor increases the sensitivity of MM cells to TMZ in vitro and in vivo. (A) A375 cells were treated with TMZ and/or vMF (0.02 and 0.04 μM) for 24 and 48 h. The growth inhibition rate of A375 cells (*p < 0.05 and **p < 0.01 compared with the TMZ group). (B) Sk‐MEL28 cells were treated with TMZ and/or vMF (0.02 and 0.04 μM) for 24 and 48 h. The growth inhibition rate of Sk‐MEL28 cells (*p < 0.05 and **p < 0.01 compared with the TMZ group). (C, D) The apoptosis rate of A375 cells treated with vMF (0.5 and 1.0 μM) and TMZ (2 mM), alone or in combination, were analysed using flow cytometry after PI/Annexin V staining (*p < 0.05 and **p < 0.01 compared with the indicated treatment). The experiments were repeated 3 times independently. (E) MM growth curve in nude mice (left panel) (*p < 0.05 and **p < 0.01 compared with the control group). (F) Average tumour weight of each group (*p < 0.05 and **p < 0.01 compared with the control group). (G) Representative images of subcutaneously transplanted MM in nude mice.

The inhibition rates of A375 and Sk‐MEL28 cells treated with various concentrations of TMZ were determined using the MTT assay to confirm the appropriate concentration of TMZ for subsequent experiments (Figure S1). As shown in Figure 5A, with increasing TMZ concentration and application time, the inhibition rates of A375 and SK‐MEL28 cells gradually increased. In other words, TMZ inhibited cell growth in a time‐ and dose‐dependent manner. The IC_50_ values of the A375 and Sk‐MEL28 cells were significantly different (Figure S1); thus, the TMZ concentration selected for the subsequent MTT assay was different. Seven data points of 0.3, 0.6, 0.9, 1.2, 1.5, 1.8 and 2.1 (mM) and 6 data points of 0.1, 0.2, 0.4, 0.8, 1.6 and 3.2 (μM) were used in A375 cells to determine the dose‐effect curve of TMZ and vMF. Different concentrations of TMZ and vMF (0.02 and 0.04 μM) were used in combination analysis (Figure S2A). Seven data points of 0.5, 1.0, 1.5, 2.0, 2.5 and 3.0 (mM) and 6 data points of 0.1, 0.2, 0.4, 0.8, 1.6 and 3.2 (μM) were used in Sk‐MEL28 cells to determine the dose‐effect curve of TMZ and vMF. Different concentrations of TMZ and vMF (0.02 and 0.04 μM) were used in combination analysis (Figure S2B). The inhibition of cell growth was evaluated with the MTT assay to confirm whether vMF increased the chemosensitivity of MM cells to TMZ. Briefly, A375 and SK‐MEL28 cells were treated with different concentrations of vMF (0.02 and 0.04 μM) and/or TMZ (0.3, 0.6, 0.9, 1.2, 1.5, 1.8 and 2.1 mM or 0.5, 1.0, 1.5, 2.0, 2.5 and 3.0 mM, respectively) for 24 h or 48 h (Figure 5A,B). Compared to TMZ alone, combined treatment with vMF increased the cell growth inhibition rate. The results of the MTT assay suggested that TMZ significantly decreased the proliferation of A375 and Sk‐MEL28 cells and that vMF increased sensitivity to TMZ‐induced cytotoxicity. A375 cells were treated with vMF (0.5 or 1 μM) and TMZ (2 mM) for 24 h either alone or in combination, and the number of apoptotic cells was detected by performing PI/Annexin V‐FITC staining (Figure 5C,D). The apoptosis rate increased from 3.37% ± 3.00% in the control group to 28.83% ± 4.02% in the 2 mM TMZ group. Compared with treatment with 2 mM TMZ alone, treatment with 2 mM TMZ in combination with 0.5 μM or 1 μM vMF increased the apoptosis rates to 41.37% ± 3.96% and 55.53% ± 5.09%, respectively. The apoptosis rates of the 1 μM vMF group and the control group were not significantly different (*p* > 0.05). The apoptosis assay indicated that vMF increased TMZ‐induced apoptosis in a dose‐dependent manner. In addition, after treatment, the tumour length and width in nude mice were surveyed every 2 days, and the tumour volume was calculated. The growth curve of A375 tumours in nude mice was drawn with time (day) as the abscissa and tumour volume (cm^3^) as the ordinate (Figure 5E). Compared with the control group, tumour growth in the TMZ and TMZ + vMF groups was significantly slower (*p < 0.05 and **P < 0.01), and the tumour growth in the TMZ + vMF group was slower than that in the TMZ group (*P* < 0.05). After 21 days of treatment, all groups of nude mice were euthanised, and the xenograft tumours were removed and then weighed (Figure 5E). The tumour weights in the TMZ group and TMZ + vMF group were significantly lower than those in the control group (*p* < 0.05 and **p < 0.01), and the tumour weight in the TMZ + vMF group was also lower than that in the TMZ group (*p* < 0.05) (Figure 5F,G). Based on these results, vMF increases the sensitivity of MM to TMZ in vivo and in vitro.

### 3.6 ERK Inhibitor Increases TMZ‐Induced DNA Damage in Melanoma

**FIGURE 6:**
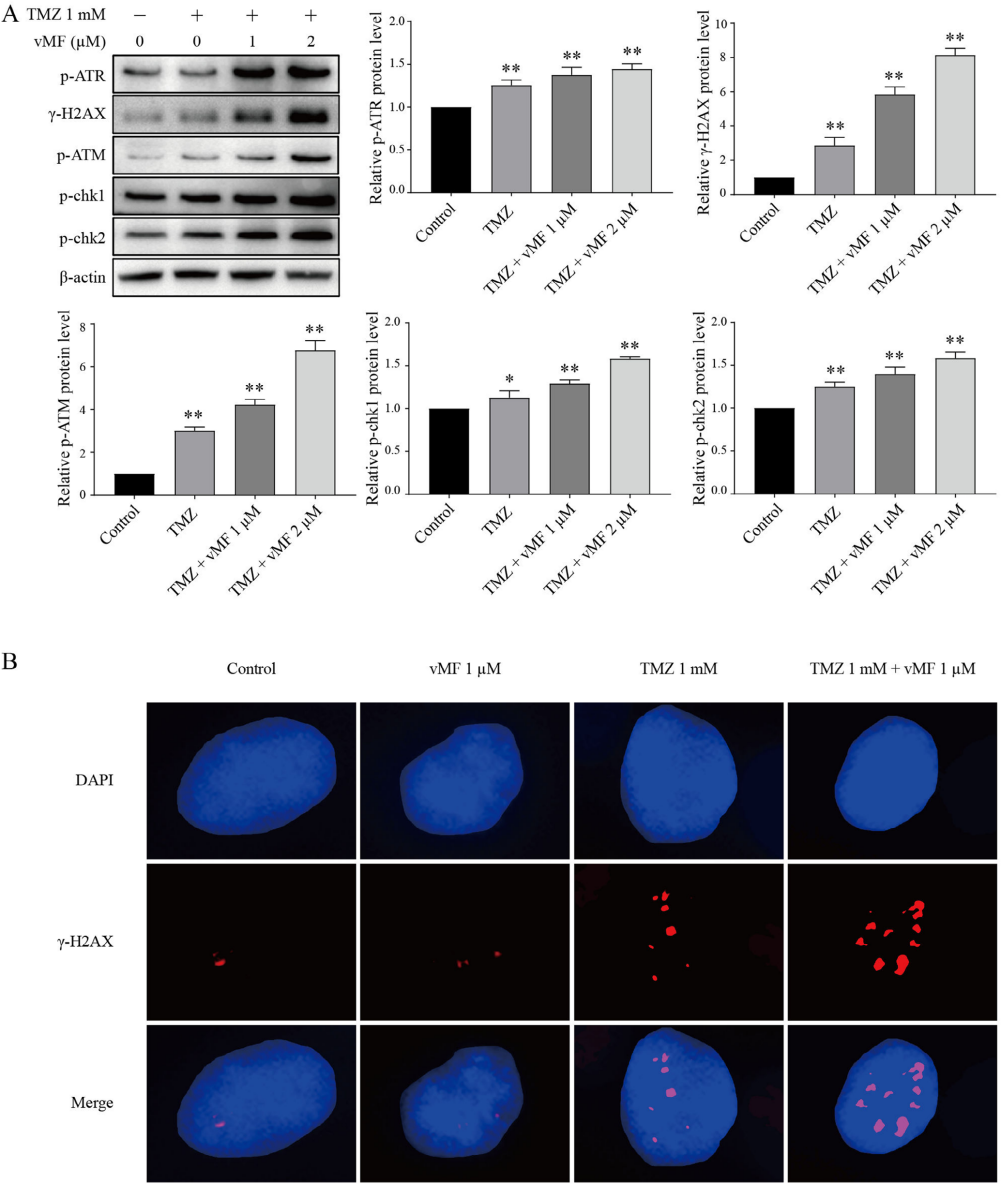
ERK inhibitor increases TMZ‐induced DNA damage in melanoma. (A) Levels of the p‐ATR, γ‐H2AX, p‐ATM, p‐Chk1 and p‐Chk2 proteins were detected in A375 cells treated with TMZ (1 mM) and vMF (1 and 2 μM) for 24 h using Western blot analysis (*p < 0.05 and **p < 0.01 compared with the control group). (B) Measurement of DNA damage in A375 cells treated with TMZ (1 mM) and/or vMF (1 μM) for 24 h. Immunofluorescence staining for γ‐H2AX (red) in A375 cells. DAPI (blue) was used to stain nuclei (representative fields at 200× magnification are shown).

The levels of the p‐ATR, γ‐H2AX, p‐ATM, p‐Chk1 and p‐Chk2 proteins were detected in A375 cells treated with vMF (1 and 2 μM) and/or TMZ (1 mM) using Western blot analysis (Figure 6A). Western blotting showed markedly higher levels of the p‐ATR, γ‐H2AX, p‐ATM, p‐Chk1 and p‐Chk2 proteins in the TMZ group, TMZ + 1 μM vMF group and TMZ + 2 μM vMF group than in the control group (*p < 0.05 and **p < 0.01). Furthermore, protein expression in the TMZ group, TMZ +1 μM vMF group and TMZ +2 μM vMF group increased sequentially. The Western blotting results indicated that vMF increased TMZ‐induced DNA damage in a dose‐dependent manner. Immunofluorescence staining was used to detect the effect of vMF on the level of the TMZ‐induced DNA damage protein γ‐H2AX (Figure 6B). Immunofluorescence staining results suggested sequential increases in γ‐H2AX levels in the control group, 1 mM TMZ group and 1 mM TMZ + 1 μM vMF group. No obvious difference was detected between the 1 μM vMF group and the control group. Thus, vMF increases the DNA damage caused by TMZ.

## 4 Discussion

In recent decades, the incidence of MM has been increasing at a rate of 6%–7% annually, and the age of patients shows a younger trend. Patients with advanced MM progress rapidly and experience a poor prognosis. Systemic therapy (chemotherapy, targeted therapy and immunotherapy) is the primary option for prolonging their survival. Chemotherapy is still the strong recommendation in the treatment of advanced malignant melanoma, and the commonly used chemotherapy drugs are dacarbazine and temozolomide, although targeted therapy has made some breakthrough progress. Tumour cells have many mechanisms of resistance to alkylating drugs, such as DNA repair proteins, cytokines and RIP2. One primary mechanism underlying TMZ resistance is the DNA repair system, which is involved in the repair of the DNA damage caused by TMZ and chloroethyl nitrosoureas (CNUs) [30, 31, 32]. O^6^‐methylguanine‐DNA methyltransferase (MGMT) is the most crucial mediator for repairing DNA damage caused by TMZ. Among DNA repair systems, MGMT‐mediated repair is unique relative to many complex DNA repair mechanisms. First, MGMT works alone and does not depend on any other proteins or cofactors. It transfers alkyl groups to internal cysteine residues, acting as both a transferase and a receptor for alkyl groups. Next, MGMT irreversibly inactivates itself when it receives alkyl groups from guanine, and thus, it is a suicidal protein. Finally, MGMT restores DNA damage stoichiometrically. Strategies that adjust MGMT activity might increase the efficacy of chemotherapy using alkylating agents [14]. Since MGMT possesses an efficient repair function for DNA damage caused by alkylating agents, MGMT expression has been employed as an index to predict the sensitivity of TMZ in GBM [33, 34]. 30%–60% of patients with GBM presenting MGMT gene silencing induced by hypermethylation in the promoter region are more likely to benefit from alkylating agent‐based chemotherapy to achieve longer survival times [35, 36]. As an important DDR protein, MGMT is widely expressed in normal human tissues but is generally expressed at high levels in all types of human tumours, including melanoma, lung cancer, glioma, leukaemia, lymphoma and colon cancer [37]. For a long time, it was believed that the MGMT promoter is mainly regulated by methylation epigenetic modification, but our research shows that the expression of MGMT could be affected at the transcription level after chemotherapy. Our data showed that TMZ stimulation could activate the ERK signalling pathway in A375 cells, which in turn increased the promoter transcriptional activity of MGMT, indicating that after TMZ treatment, intracellular MGMT depletion increased and reserves decreased, but MGMT transcription could be enhanced. Therefore, we believe that the transcription of the MGMT promoter could be blocked by inhibiting the ERK pathway to reduce the level of MGMT protein, thus antagonising the drug resistance of tumour cells to TMZ.

The experimental results illustrated a decrease in the total amount of MGMT protein after TMZ treatment, indicating that TMZ treatment consumed a large amount of MGMT protein to repair damage after alkylating agent stimulation, but the ERK signalling pathway was activated at this time (Figure 2A). The activated ERK signalling pathway promotes the transcription of MGMT to compensate for the depleted MGMT protein. However, when the ERK pathway was blocked by vMF at the same time, the compensation of MGMT protein was insufficient (Figure 2C), which was disadvantageous to the DNA repair of tumour cells.

Moreover, we analysed MGMT gene expression and survival‐related data in skin cutaneous malignant melanoma (SKCM) on the GEPIA online site (http://gepia.cancer‐pku.cn/) and found that MGMT expression was lower in SKCM than in adjacent tissues (Figure S3A). This further echoed our experimental results that the transcription of MGMT was not high in melanoma in the absence of drugs but could be strengthened compensatively after exposure to TMZ. The promotion of MGMT transcription after TMZ action may be a mechanism of acquired drug resistance in melanoma cells. There was no significant difference in overall survival (OS) between patients with low MGMT expression and those with high MGMT expression (Figure S3B), which may be related to the development of acquired drug resistance after drug treatment of melanoma cells. However, the mechanism of acquired drug resistance is unclear. In the past, we considered that the mechanism of TMZ resistance is mainly due to the expression level of MGMT. The density of methylated CpG near the promoter was the main determinant of repression. The weak promoter could be completely repressed by the dispersed methylated CpG, and the transcription stopped completely, which was why many studies have suggested that patients with MGMT promoter methylation have better efficacy with TMZ in glioblastoma [37]. Although MGMT level was not high in MM cells with chemotherapy, the transcriptional activity of MGMT could be improved by activation of the ERK signalling pathway after TMZ treatment, which may be one of the mechanisms of resistance to TMZ malignant tumour. Therefore, we confirmed that the mechanism of acquisition of TMZ in melanoma is mainly through affecting the transcription of MGMT.

TMZ is a common alkylating agent used to treat advanced melanoma. A large phase III clinical study indicated that TMZ was more efficient than DTIC in advanced MM [4]. Some studies have shown that ERK phosphorylation is elevated after treatment with TMZ [38]. Meanwhile, MGMT transcriptional activity also decreased correspondingly after the use of the ERK inhibitor U0126, suggesting that TMZ may promote the transcription of MGMT by regulating related transcription factors via activation of the ERK pathway in malignant melanoma cells. Our experiments also found that the level of p‐ERK1/2 in MM cells increased after TMZ treatment. We postulate that this could be the underlying mechanism through which TMZ enhances the transcriptional activity of the promoter. Since the ERK inhibitor vMF was approved by the FDA in 2011 and has since been widely applied in the clinical management of MM, this oral targeted drug is rarely discontinued due to adverse reactions, which helps to elucidate the mechanism of MGMT inhibitors used as TMZ sensitisation agents. vMF reversibly and highly selectively binds the ATP‐binding domain of the mutant BRAF monomer [39] and reduces ERK1/2 phosphorylation and cyclin D1 levels, which may suppress cell proliferation [40]. At the same time, we found that the ERK inhibitor vMF downregulates the expression of MGMT in the A375 and Sk‐MEL28 melanoma cell lines, indicating that the ERK inhibitor vMF may increase TMZ cytotoxicity in melanoma cells. In a previous report by Sato et al., MEK inhibitors reversed the resistance of glioblastoma cells to TMZ, and MEK inhibitors combined with TMZ effectively controlled the occurrence and development of glioblastoma cells. A strategy targeting the MEK–ERK–MDM2–p53 pathway combined with TMZ may be a novel and promising treatment for glioblastoma [41]. Although related studies have assessed the molecular mechanism regulating MGMT in glioblastoma, the signal transduction pathway regulating MGMT in melanoma cells has not been elucidated. Within the scope of our investigation, we explored signalling pathways that regulate MGMT expression in melanoma cells and found that TMZ stimulation promoted p‐ERK1/2 protein expression and increased MGMT promoter transcriptional activity. This could potentially elucidate the mechanism underlying the acquisition of drug resistance. We further confirmed whether vMF functions via the ERK–MGMT pathway by performing an analysis that revealed that high expression of MGMT reversed the effect of ERK inhibitors on sensitising cells to TMZ. This result suggests that highly expressed melanoma cells are still resistant to chemotherapy even with the use of inhibitors. The specific mechanism needs to be further studied.

MEK/ERK signals are constitutively activated due to the upregulation or abnormalities of upstream molecules of tyrosine kinase receptors (EGFR, PDGFR, etc.) [42, 43]. Previous research investigating the association between MEK/ERK signalling and MGMT expression levels illustrated that the MEK inhibitors U0126 and SL327 inhibited MGMT expression in GSCs by activating p53. MGMT inhibitors inhibit the expression of MGMT and regulate GSC resistance to TMZ [44]. Based on this evidence, therapies targeting MEK/ERK signalling might be a potential option for the treatment of TMZ‐resistant and TMZ‐naive cases. The relationship between MAPK/ERK pathway activity and MGMT expression in MM tissue has not been clearly defined in the literature. Notably, our in vitro experiments showed that activation of the ERK pathway in cells was further increased after TMZ exposure, promoting the transcription of MGMT. This finding may represent the protective response of cells to toxic drugs, which may further mediate drug resistance. Our data indicated clinical prospects for ERK inhibitor sensitisation strategies.

Our study has demonstrated that one of the mechanisms by which tumour cells develop resistance to drugs is through TMZ promoting the transcription of MGMT, which is achieved by activating the ERK pathway. Therefore, we propose that inhibiting this chemoresistance mechanism can be achieved through the combination of chemotherapy and ERK inhibitors.

## 5 Conclusions

Our experimental findings suggest that TMZ promotes the transcription of MGMT by activating the ERK signalling pathway and that the use of an ERK pathway inhibitor effectively increases the sensitivity of melanoma cell lines to TMZ both in vivo and in vitro by downregulating MGMT levels. Furthermore, we observed a positive correlation between the levels of MGMT and p‐ERK1/2 proteins. Based on these results, we propose that the combination of an ERK pathway inhibitor and TMZ represents a promising therapeutic strategy for the clinical treatment of MM.

## Author Contributions

**Meiyi Deng:** data curation (equal). **Bingjie Ren:** formal analysis (equal). **Jing Zhao:** data curation (equal). **Xia Guo:** methodology (equal). **Yuanyuan Yang:** data curation (equal). **Huiling Shi:** methodology (equal). **Xuyu Bian:** formal analysis (equal). **Mengyao Wu:** formal analysis (equal). **Caihua Xu:** resources (equal). **Min Tao:** conceptualization (equal). **Rongrui Liang:** conceptualization (equal). **Qiang Li:** conceptualization (equal).

## Ethics Statement

The animal protocol was approved by the Dushu Lake Hospital Affiliated to Soochow University (Soochow University, China).

## Consent

The authors have nothing to report.

## Conflicts of Interest

No financial and personal relationships with other people or organisations could inappropriately influence (bias) this work.

## Supporting information


**Figure S1.** TMZ inhibits MM cell growth in a time‐ and dose‐dependent manner. (A) A375 cells showed a decreased growth rate. (B) SK‐MEL28 cells showed a decreased growth rate. (Cells were seeded into 96‐well plates, and cell growth inhibition rate was calculated. Data from multiple experiments are expressed as the mean ± SD.).

**Figure S2.** Combination analysis of different concentrations of TMZ and vMF at different times. (A) The combination of TMZ and vMF shows an additive effect in A375 cells. (B) The combination of TMZ and vMF shows a synergy effect in SK‐MEL28 cells.

**Figure S3.** MGMT gene expression and survival‐related data in SKCM. (A) MGMT expression levels in SKCM. (B) Overall survival in SKCM with different MGMT expression profiles.

## Data Availability

The original contributions presented in the study are included in the article/Supporting Information. Further inquiries can be directed to the corresponding authors.
