# Supplementary figures and images for: Temozolomide‐Promoted MGMT Transcription Contributes to Chemoresistance by Activating the ERK Signalling Pathway in Malignant Melanoma
Source: J Cell Mol Med. 2025 Jan 28;29(3):e70380. doi: 10.1111/jcmm.70380 (PMC11773391; doi:10.1111/jcmm.70380)

A

A375

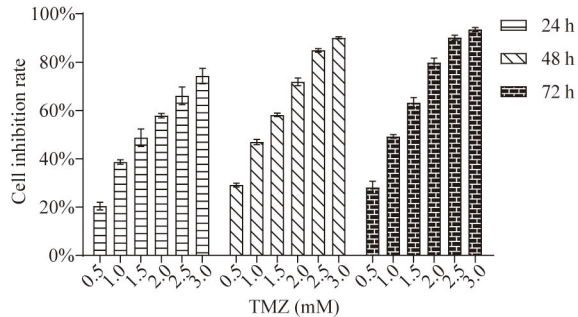

B

SK-MEL28

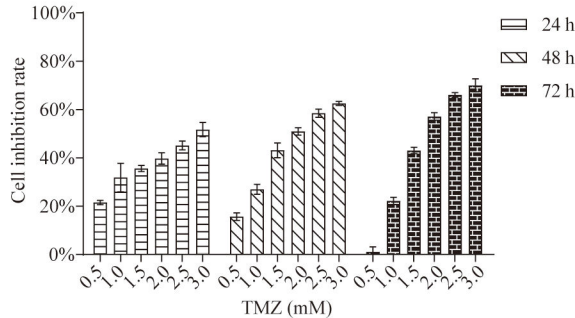

Supplement: Supplementary file 1 — Figure S1. TMZ inhibits MM cell growth in a time‐ and dose‐dependent manner. (A) A375 cells showed a decreased growth rate. (B) SK‐MEL28 cells showed a decreased growth rate. (Cells were seeded into 96‐well plates, and cell growth inhibition rate was calculated. Data from multiple experiments are expressed as the mean ± SD.). [file JCMM-29-e70380-s001.pdf]

A

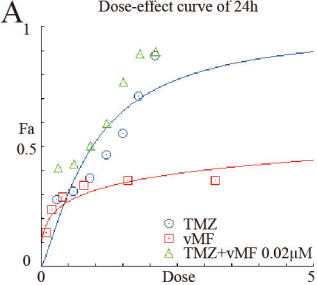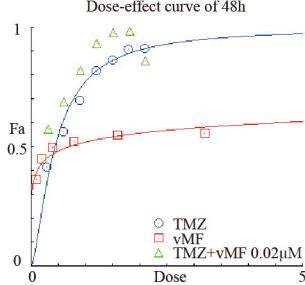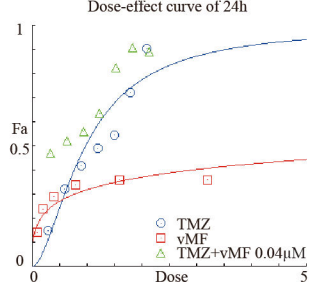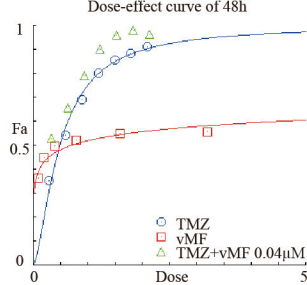

B

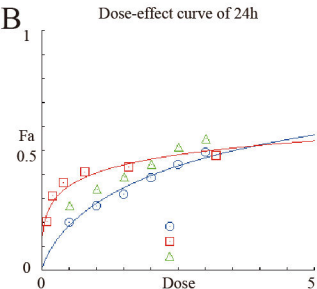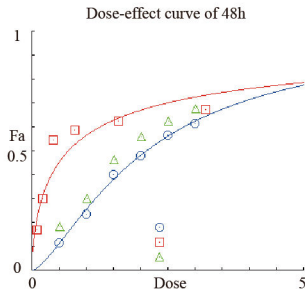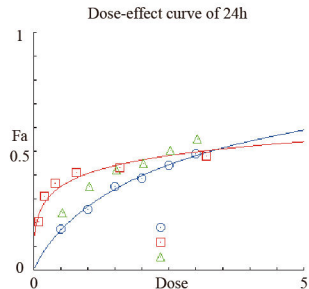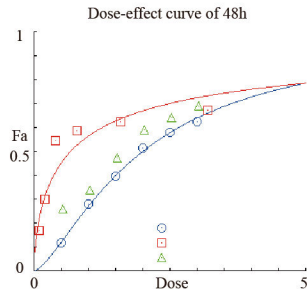

Supplement: Supplementary file 2 — Figure S2. Combination analysis of different concentrations of TMZ and vMF at different times. (A) The combination of TMZ and vMF shows an additive effect in A375 cells. (B) The combination of TMZ and vMF shows a synergy effect in SK‐MEL28 cells. [file JCMM-29-e70380-s002.pdf]

A

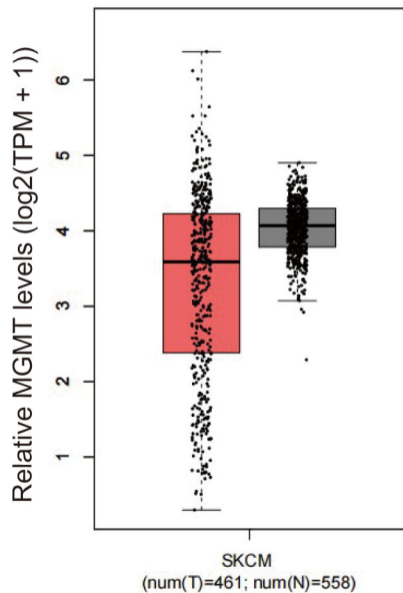

B

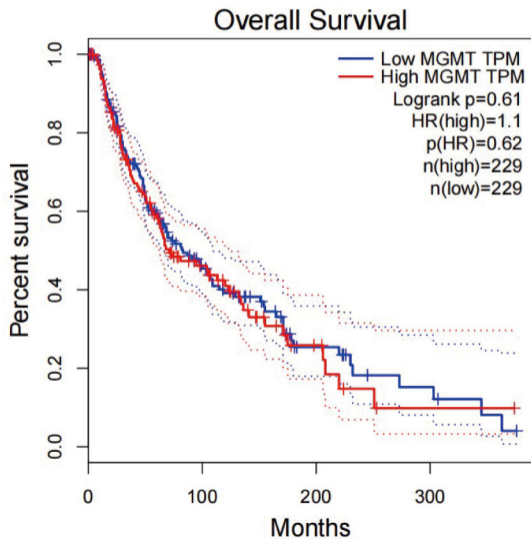

Supplement: Supplementary file 3 — Figure S3. MGMT gene expression and survival‐related data in SKCM. (A) MGMT expression levels in SKCM. (B) Overall survival in SKCM with different MGMT expression profiles. [file JCMM-29-e70380-s003.pdf]
